# The taxonomic name resolution service: an online tool for automated standardization of plant names

**DOI:** 10.1186/1471-2105-14-16

**Published:** 2013-01-16

**Authors:** Brad Boyle, Nicole Hopkins, Zhenyuan Lu, Juan Antonio Raygoza Garay, Dmitry Mozzherin, Tony Rees, Naim Matasci, Martha L Narro, William H Piel, Sheldon J Mckay, Sonya Lowry, Chris Freeland, Robert K Peet, Brian J Enquist

**Affiliations:** 1 Department of Ecology and Evolutionary Biology, University of Arizona Tucson, P.O. Box 210088, Tucson, AZ, 85721, USA; 2 The iPlant Collaborative, Thomas W. Keating Bioresearch Building, 1657 East Helen Street, Tucson, AZ, 85721, USA; 3 BIO5 Institute, 1657 East Helen Street, PO Box 210240, Tucson, AZ, 85721-0240, USA; 4 Cold Spring Harbor Laboratory, 1 Bungtown Road, Cold Spring Harbor, NY, 11724-2202, USA; 5 7 MBL street, Center for Library and Informatics, Marine Biological Laboratory, 7 MBL Street, Woods Hole, MA, 02543, USA; 6 Divisional Data Centre, CSIRO Marine and Atmospheric Research, GPO Box 1538, Hobart, Tasmania, 7001, Australia; 7 Yale-NUS College, 6 College Avenue East, Singapore, 138614, Singapore; 8 Missouri Botanical Garden, 4344 Shaw Blvd. |, St. Louis, MO, 63110, USA; 9 Department of Biology, CB 3280, University of North Carolina, Chapel Hill, NC, 27599-3280, USA; 10 The Santa Fe Institute, 1399 Hyde Park Road, Santa Fe, NM, 87501, USA

**Keywords:** Biodiversity informatics, Database integration, Taxonomy, Plants

## Abstract

**Background:**

The digitization of biodiversity data is leading to the widespread application of taxon names that are superfluous, ambiguous or incorrect, resulting in mismatched records and inflated species numbers. The ultimate consequences of misspelled names and bad taxonomy are erroneous scientific conclusions and faulty policy decisions. The lack of tools for correcting this ‘names problem’ has become a fundamental obstacle to integrating disparate data sources and advancing the progress of biodiversity science.

**Results:**

The TNRS, or Taxonomic Name Resolution Service, is an online application for automated and user-supervised standardization of plant scientific names. The TNRS builds upon and extends existing open-source applications for name parsing and fuzzy matching. Names are standardized against multiple reference taxonomies, including the Missouri Botanical Garden's Tropicos database. Capable of processing thousands of names in a single operation, the TNRS parses and corrects misspelled names and authorities, standardizes variant spellings, and converts nomenclatural synonyms to accepted names. Family names can be included to increase match accuracy and resolve many types of homonyms. Partial matching of higher taxa combined with extraction of annotations, accession numbers and morphospecies allows the TNRS to standardize taxonomy across a broad range of active and legacy datasets.

**Conclusions:**

We show how the TNRS can resolve many forms of taxonomic semantic heterogeneity, correct spelling errors and eliminate spurious names. As a result, the TNRS can aid the integration of disparate biological datasets. Although the TNRS was developed to aid in standardizing plant names, its underlying algorithms and design can be extended to all organisms and nomenclatural codes. The TNRS is accessible via a web interface at http://tnrs.iplantcollaborative.org/ and as a RESTful web service and application programming interface. Source code is available at https://github.com/iPlantCollaborativeOpenSource/TNRS/.

## Background

The past two decades have seen an explosive growth of biodiversity databases, providing access to millions of species observations. The more prominent large databases include compilations of museum records and observations (e.g., GBIF [1], Tropicos [2], REMIB [3], OBIS [4], VertNet [5], MaNIS [6]), fossil datasets (The Paleobiology Database [7]), ecological inventories (VegBank [8], SALVIAS [9], USFS FIA database [10], Forest Plots Database [11], CTFS [12]; see GIVD [13]), trait measurements (TraitNet [14], TRY [15]), molecular sequences (GenBank [16]) and phylogenies (TreeBase [17]). Collectively, these databases encompass hundreds of thousands of species [18]. This vast and growing information resource is being used to address fundamental questions in ecology, evolution and systematics [17,18] and to explore patterns in the distribution of organismal form, function and diversity at previously impossible temporal and spatial scales [19-23]. Researchers are only beginning to explore the potential applications of these global biodiversity data sources for agriculture [20], plant products research [21] and conservation biology [22]. Integration of such large, disparate, and heterogeneous datasets has involved overcoming numerous challenges of data exchange, interoperability, and scaling [18,24]. Despite considerable progress [25], however, one critical challenge remains largely unsolved: the correction and standardization of taxonomic names in scientific data and literature.

Incorrect, ambiguous or synonymous taxon names present a fundamental problem for the study of comparative biology and biodiversity [26]. Ecological studies that encompass large numbers of species, conservation decisions based on data from many sources, and phylogenetic analyses linking sequence data to phenotypic traits all require accurate matching of species identities among datasets. If uncorrected, lack of standardization of species names can result in mismatched observations and inflated measures of species richness, leading to erroneous scientific conclusions, faulty conservation policy, and an inability to make reliable predictions across space and time [27]. Although progress has been made toward developing an authoritative global taxonomy (Global Names [28], The Plant List [24]), the growing availability of digitized sources of names (International Plant Names Index [29], Global Names [30], Tropicos [2], ZooBank [31], UBio [32], Encyclopedia of Life [33], Integrated Taxonomic Information System [34], Catalogue of Life [35]), identifiers (Global Names [30], UBio [31], ZooBank [32]) and taxonomic opinion (Tropicos [2], The Plant List [36]), has yet to provide a solution to the rapid accumulation of erroneous names in the scientific literature and data repositories.

Recent applications for the automated recognition of taxon names [34] have accelerated the digitization of biodiversity literature [35]. Unfortunately, the inability of these applications to recognize and correct ambiguous or erroneous scientific names means they fall short of meeting the needs of researchers. Combining large datasets from different sources requires careful standardization of hundreds or thousands of taxon names—a task that must be performed manually or with *ad hoc* scripting, resulting in duplication of effort and propagation of error. In short, the lack of automated tools and standardized workflows for correcting taxonomic names is a major impediment to conducting synthetic science with heterogeneous sources of biodiversity data [37].

How widespread is taxonomic error? A recent study of New World plant distributions and species richness [38] illustrates the severity of the problem. Compilation of 308,000 geo-referenced plant observations from 51 digitized sources of herbarium specimens and forest inventories resulted in 22,100 unique species names; after correcting misspellings and updating synonymous names, that total was reduced to 12,980 accepted species. Thus, over 42% of the names in the original data were erroneous, obsolete, or otherwise inconsistent with currently accepted names. Uncritical use of the original, uncorrected taxon names would have grossly inflated species richness and led to distorted, possibly biased distributional patterns due to spurious species with artificially small ranges. At best, erroneous taxon names limit the usefulness of the data they mislabel by preventing linkages among observations of the same organism; at worst, they represent an insidious source of error.

Misspelled names are just one component of the larger problem of taxonomic semantic heterogeneity [39]. Such ambiguity can arise for a number of reasons: (1) misspellings, vernacular variants, and lexical variants (different ways of writing the same name); (2) homotypic synonyms (sets of different scientific names based on the same type specimen and representing changes in genus classification or technical changes such as substitute names, that objectively refer to the same taxon); (3) heterotypic synonyms (names that may or may not refer to the same taxon, depending on expert opinion); (4) homonyms (identical names that refer to different taxa); and (5) differing taxonomic concepts (narrower or broader interpretations of taxa represented by the same name and authority [39]). While automated and semi-automated applications can frequently address the first and second sources of error, the third, fourth and fifth present significantly more difficult challenges. For example, resolution of complex, or *pro parte*, synonyms (for example, a species which was split into two or more species) requires additional information such as when and where the name was used. Disambiguating homonyms requires information on higher taxa such as family or kingdom (although homonyms in the same family can only be distinguished by the authorities portions of the scientific names). Even if a name is correctly resolved to an accepted taxon, the exact circumscription of that taxon can vary from expert to expert; such taxon concepts are not easily or precisely communicated by names alone [39]. While no automated system can perfectly resolve all the kinds of taxonomic problems listed, a service that corrects variant and erroneous spellings, disambiguates homonyms by means of higher taxonomic filtering, and updates simple synonyms with reference to authoritative taxonomic sources would go a long way toward solving the "names problem". Here we present such a solution, the Taxonomic Name Resolution Service.

## Implementation

### Overview

The Taxonomic Name Resolution Service, or TNRS, is an application for automated and user-supervised correction and standardization of plant taxonomic names. Developed by the iPlant Collaborative [40] as a collaboration between the iPlant Tree of Life project [41] and the Botanical Information and Ecology Network [42], the TNRS standardizes names according to one or more authoritative taxonomic sources. Capable of processing thousands of names in a single operation, the TNRS detects likely misspelled taxon names, transforms names and authorities to a single canonical form, converts synonyms to accepted names, discriminates among many types of homonyms, and detects and flags ambiguous results. The TNRS also handles features peculiar to both ecological data (such as morphospecies and partial identifications) and phylogenetic data (such as embedded accession codes). The TNRS is accessible both as a web service and a user-friendly web interface.

Four core principles guided the development of the TNRS. First, use existing sources of high-quality, digitized taxonomy that provide information on synonymy in addition to names. Second, build on existing applications whenever possible. Third, use Open Source tools and adhere to Open Source principles [43], including public release of all source code. Fourth, provide a generalizable solution extendable to other organisms and nomenclatural codes—not just plants.

### Taxonomic sources

The TNRS resolves names against a local cache of external taxonomic sources (see ***The TNRS database***, below). Currently, the default taxonomic sources used by the TNRS are the Missouri Botanical Garden's Tropicos database [2], the Global Compositae Checklist [44] and USDA Plants [45] (Table 1); users select one of these sources as a standard against which to standardize their names. Combining sources is also possible; this should be done with caution due to different spelling conventions and potentially conflicting synonymies. A partial solution to such conflicts is to assign priority to each source, such that a second source is consulted for a particular name only if that name cannot be matched using the first source (see ***User options***, below). NCBI taxonomy [46] (Table 1) is also provided as an optional source for users wishing to match their names to taxa with molecular sequence data in GenBank [16]. However, due to missing taxa, inconsistent taxonomy and the presence of numerous informal names or "dark taxa" [47], users are cautioned against using NCBI for taxonomic standardization.

**Table 1:** Details of taxonomic sources used by the TNRS

**Name**	**Total names**	**Taxonomic scope**	**Geographic scope**	**Primary URL**
Tropicos	1,250,897	Embryophytes	Comprehensive coverage of North, Central and South America; partial coverage of Old World, especially Madagascar, Aast Africa and China.	http://www.tropicos.org/
USDA Plants	93,307	Embryophytes and lichens	U.S. and its territories, Canada, Greenland	http://plants.usda.gov/java/
Global Compositae Checklist	123,551	Asteraceae	Global	http://compositae.landcareresearch.co.nz/
NCBI Taxonomy	210,214	Embryophytes	Global	http://www.ncbi.nlm.nih.gov/taxonomy

Total names includes higher taxa and infraspecific taxa in addition to species. Taxonomic scope refers to the subset of the database used for the TNRS (for example, NCBI Taxonomy covers the entire tree of life, not just embryophytes). "Embryophytes" are flowering plants, conifers, ferns, mosses, hornworts and liverworts.

Taxonomic sources currently accessed by the TNRS provide nearly complete coverage of land plants (mosses, liverworts, hornworts, ferns, lycophytes, gymnosperms and flowering plants) for the New World (Table 1). With the exception of the flowering plant family Asteraceae, coverage of Old World plant names is less complete. A central goal of the TNRS is to enable users to resolve names of all organisms governed by the International Code of Nomenclature for algae, fungi and plants (ICN) [48], and we invite curators of high quality taxonomic databases to help fill gaps in our current taxonomic coverage by exposing their content via the iPlant TNRS. The TNRS website provides information on how to become a data provider to the TNRS, including a description of a simple exchange schema which can be used to expose taxonomic content to automatic validation and ingest by the TNRS (see http://tnrs.iplantcollaborative.org/sources.html#Provider). Alternatively, taxonomic data providers can deploy their own instance of the TNRS using source code available from the iPlant OpenSource repository on GitHub (see https://github.com/iPlantCollaborativeOpenSource/TNRS/).

### Components

The TNRS consists of four main components: (1) the TNRS database, which contains names and synonymy from external taxonomic sources; (2) a name resolution engine consisting of a name parsing application and a fuzzy matching application; (3) a web services layer and application programming interface (API); and (4) a web-based user interface.

#### The TNRS database

The TNRS database is a periodically refreshed local cache of external sources of taxonomy, and consists of two interrelated components: (1) a MySQL core database containing the normalized and indexed names, synonymy and higher classifications, and (2) partially denormalized representations of the same taxonomic content, optimized for use by the fuzzy matching application. Information stored in the core database includes names and authors, an indication of taxonomic rank, a pointer to the immediate parent within the taxonomic hierarchy, and assertions as to the validity of a name (e.g., "accepted", "not accepted") accompanied by a pointer to the accepted name for synonymous names.

Parent-child links and synonym-accepted name assertions are stored separately from the names themselves, thus allowing storage of multiple classifications and taxonomic opinions. Retrieval of ancestor and descendent taxa to arbitrary depth is supported by secondary indexing according to a modified preorder tree traversal algorithm [49]. Two sources (Tropicos, equivalent to the APG III classification [50], and NCBI taxonomy) serve as alternative family classifications; genera, species and infraspecific taxa from all sources are joined to these families by genus.

Taxonomic content is normalized to the source database by loading scripts written in PHP. The normalization process separates names from classifications and assertions of synonymy, joining new names to alternative family classifications and building foreign keys and indexes. The loading scripts also pre-load the fuzzy matching tables and perform critical validations such as checking for missing or conflicting parent-child links. Taxonomic content can be exposed to the TNRS using an exchange schema based on Simple Darwin Core [51]. For details of the "TNRS Simple Darwin Core Format" see http://tnrs.iplantcollaborative.org/sources.html#DWC.

#### Name resolution engine

Name resolution by the TNRS consists of four steps: pre-processing, name parsing, fuzzy matching and post-processing.

#### Pre-processing

Prior to submitting names to the parsing and fuzzy-matching applications, family names pre-pended to species names are removed by searching the initial string of the name for standard family endings (“aceae” and “idae”) and checking against a list of conserved plant family names (Gramineae, Compositae, etc.; although plant-specific, the latter check could be generalized by expanding this list to include conserved family names from all nomenclatural codes). Indications of uncertain identification such as “cf.” and “aff.” [52] are also removed. For names submitted as all capital letters, case is adjusted by capitalizing the first letter and setting all remaining letters to lower case. This last step is necessary as the name parser uses case to identify name components and cannot correctly parse all-caps names.

The final step in pre-processing is to match the remaining string directly against the core database. Strings matching completely are given an overall match score of 1.0 (see ***Match score calculation***) and removed from further processing. Unmatched names are passed to the name parser (see ***Name parsing***). The results of parsing are matched a second time against the core database before passing the remaining unmatched names to the fuzzy matching application (see ***Fuzzy matching***).

#### Name parsing

Separation and classification of name components is performed by the GNA Scientific Name Parser [53], which is distributed as a Ruby gem library, a command line utility and a server script. It is based on Treetop gem which implements the Parsing Expression Grammars algorithm [54]. The parser defines the components of a scientific name as a series of recursive regular expressions. It begins by using white spaces to separate the components of the scientific name and authorship, and then moves to identifying each components as a genus, specific epithet, infraspecific epithet, author, year, etc. The higher level definitions describe how simpler components combine together as a name or a conglomerate of names (hybrids). At first the parser follows the rules of all nomenclatural codes inclusively; if something is allowed in the ICBN (International Code of Botanical Nomenclature, but not allowed in the ICZN (International Code of Zoological Nomenclature), it is allowed by the parser. If the parser fails to atomize a name it moves into ‘relaxed’ mode, where common mistakes in writing names or authorship are taken into account. For example, relaxed mode allows diacritic characters not permitted by zoological or botanical codes, double parentheses surrounding author names, year without an author, square brackets and question marks around years (as in the example ‘[185?]’), etc. Relaxed mode does not perform fuzzy matching. If relaxed mode fails as well the parser uses ‘salvage’ mode, which tries to extract the canonical form of the name from the string, discarding anything to the right of it.` Parsing is case sensitive, which means, for example, that the genus part of a binomial must be capitalized, and the species epithet must be in lower case to be recognized. Scientific names that do not follow a rigid linear structure (for example, hybrid names such as *Coeloglossum viride* (L.) Hartman × *Dactylorhiza majalis* (Rchb. f.) P.F. Hunt & Summerhayes ssp. *praetermissa* (Druce) D.M. Moore & Soó) are also supported as a result of a recursive nature of the algorithm.

In addition to separating the author from the taxon name, the parser detects and separates the genus from specific and infraspecific epithets, and extracts rank indicators such as “var.”, “ssp.”, “subsp.”, etc. For example, “*Bromus inermis* var. *confinis* (Nees ex Steud.) Stapf” is separated into genus "Bromus", specific epithet "inermis", infraspecific rank indicator "var.", infraspecific epithet "confinis", basionym author "Nees ex Steud." and combining author "Stapf". The results of parsing are also used to determine the overall taxonomic rank of the name submitted (for example, genus, species, subspecies, variety, etc.). This information is required for flagging partial matches and for constraining matches by higher taxonomy (see ***User options***).

#### Fuzzy matching

Fuzzy matching is performed by a modified version of the PHP implementation [55] of Taxamatch [56]. The Taxamatch algorithm speeds matching of taxonomic names by matching higher taxonomic name components first, then searching only for taxa within the best-matching higher taxon (for example, genera, followed by the species within the best-matching genus). Matches to names minus the authority are determined using two separate tests: phonetic similarity and orthographic (spelling) similarity. A name passing either of these tests, or both, is considered a "match" (although see ***Candidate match selection*** for additional rules enforced by the TNRS).

Phonetic similarity is assessed using a custom algorithm that substitutes specific characters or character pairs for others, thereby transforming each name to a simplified phonetic equivalent. Although similar to approaches such as Soundex [57] and Phonix [58], the Taxamatch algorithm also takes into account specific lexical conventions of scientific names and incorporates a degree of “stemming” of species epithets, in which a range of possible variant word endings are transformed to a single standardized form (cf. [59]). The stemming (equivalent) in Taxamatch equates -a, -is -us, -ys, -es, -um, -as and -os when they occur at the end of a species epithet (or infraspecies) by changing them all to -a. Thus (for example) the epithets “nitidus”, “nitidum”, “nitidus” and “nitida” will all be considered equivalent following this process. Once transformed, names are compared using an exact match; this operation is very fast as reference names are transformed in advance during the loading of each taxonomic source to the TNRS database.

Orthographic similarity for each name component (e.g., genus, species, subspecies; but not author; see below) is calculated using a modified Damerau-Levenshtein Distance [60,61] with additional corrections for transposed syllables (T. Rees, unpubl.), hereafter referred to as edit distance (ED). “Classic” ED using Levenshtein’s original algorithm [61] is a measure of the minimum number of single-character deletions, insertions, or substitutions required to transform one string into a second string. Thus, “faveolata” and “flaveolata” have an edit distance of 1 by that measure (single character insertion) as do “Ficus” and “Fucus” (single character substitution). The “Damerau-Levenshtein” version of the algorithm also allows single character transpositions (for example, “Nais” vs. “Nias”) at a cost of ED 1 which under “classic” Levenshtein would incur a cost of 2 (substitutions), since transpositions are not recognised in the original case. The additional modification introduced for Taxamatch, termed Modified Damerau-Levenshtein Distance or MDLD, further permits multi-character transpositions (for example, “vecusilosus” to “vesiculosus”), at a cost of the number of transposed characters only (ED 2 in this case) rather than the more expensive cost (ED 4) that would be incurred if each character were to be substituted individually, as in either of the preceding algorithms.

Due to its variable spelling, abbreviation and format, similarity of the author is calculated using the more relaxed n-gram method [62], which produces an author match score (AMS) ranging from 0-1. This index is calculated as a blend of 2/3 bigram and 1/3 trigram similarity between the strings, for which known botanical author abbreviations are expanded according to a dictionary of stored abbreviations prior to the comparison. The abbreviations are chiefly a subset of the standard abbreviations found in Brummitt & Powell [63], supplemented with additional abbreviated forms, including some for animal names, as compiled in one of the authors' (TR) *Interim Register of Marine and Nonmarine Genera* database [64]. The index is calculated twice, once using the original UTF8 strings and a second time using plain ASCII version, so as to reduce differences solely due to presence or absence of diacritical marks. The final author similarity score is the unweighted average of the two calculations. For example, consider the authority portions of the two species name strings “Jovetia erecta Guédès” vs. “Jovetia erecta M.Guedes”. Treating the letters with diacritics (“é” and “è”) as different characters from their non-diacritic equivalents (“e” in both cases) would result in an undesirably low similarity (0.305, or 0.411 if the leading “M.” initial is omitted) whereas treating both as identical to “e” results in arguably too high a similarity (0.795, or 1.0 if the leading “M.” initial is omitted). Therefore, in order to score these variants as similar but not identical, the average value of the two approaches is used (0.550, or 0.705 if the leading “M.” initial is omitted). This example of multiple accented characters in a comparatively short word is somewhat unusual; in most cases the difference between the two approaches will be apparent but less extreme.

Extensions to the original Taxamatch code and schema were made to support matching of family names, trinomials (e.g., *Bromus inermis* subsp. *inermis*) and quadrinomials (e.g., *Bromus inermis* subsp. *inermis* var. *divaricatus*). An overall match score based on both name and author similarity scores is calculated during the post-processing stage (see ***Match score calculation***, below).

#### Post-processing

After fuzzy matching is complete, the following "post-processing" steps are performed: (1) calculating and scaling the overall match score, (2) applying thresholds to select the candidate best matches, (3) ranking results to select the single best match, and (4) assigning warnings. After these steps are complete, the results are returned as JSON (JavaScript Object Notation) to the web services layer.

***Match score calculation.*** After fuzzy matching is complete, the EDs of each name component (family, genus, species, variety, etc.; see ***Fuzzy matching,*** above) are combined and transformed to an Overall Match Score (OMS). The OMS provides a more intuitive measure of the confidence that a submitted string matches a name, with 0 indicating no confidence in a match (or, possibly, high confidence in a non-match) and 1 indicating certainty that the returned name is the correct match for the submitted name. With the exception of names matching perfectly to the TNRS database—which are automatically assigned an OMS of 1—calculation of the OMS involves the following four steps.

First, each name component (except author; see below) is assigned a partial match score (PMS) based on the ED between it and the closest name in the TNRS database as follows:

(1) $$\mathrm{PMS}=1-2\times \left(\mathrm{ED}/\mathrm{MaxED}\right)$$

where MaxED, the maximum possible value of ED, is equal to the length of the longest of the two strings compared. PMS thus ranges between -1 and 1, where 1 is an exact match. A penalty of -0.3 is applied if a rank indicator (“var.”, “ssp.”, “subsp.”, etc.) is present in the submitted string but is not the correct one. For example, in the case of *Chondrophora nudata* var. *virgata*, if the user submits *Chondrophora nudata* fo. *virgata*, the infraspecific taxon will receive a score of 0.7: 1 for the infraspecific epithet, minus 0.3 for the incorrect infraspecific rank indicator ("fo." instead of "var.").

**Figure 1:**
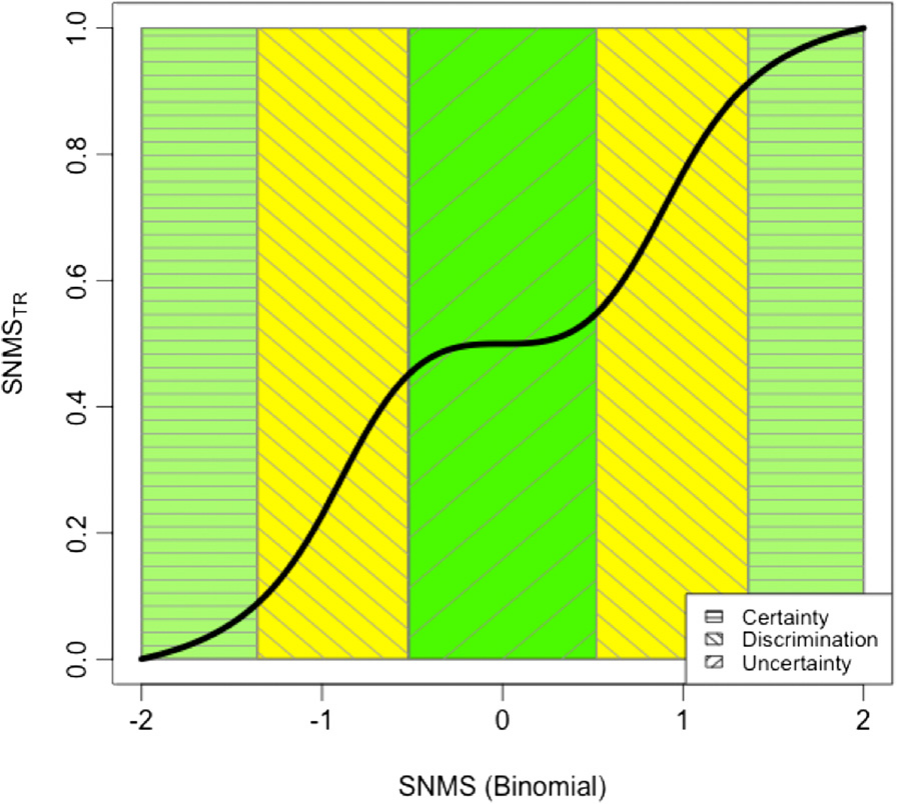
**Transformed scientific name match score (SNMS**_**tr**_**) versus original, untransformed score (SNMS) of a submitted binomial, showing the differing degrees of certainty defined by the transformation function.** In the two regions of certainty, small score differences have a smaller impact on the outcome: either there is a mismatch (SNMS=-2) or a perfect match (SNMS=2). Similarly, in the region of uncertainty, small score differences do not help to distinguish between matches and mismatches. In the regions of discrimination, instead, there is already a preference towards matches or mismatches, and small differences can help tip the balance.

The second and third steps involve calculation of the original and transformed scientific name match scores (SNMS and SNMS_tr_, respectively). SNMS is simply the sum of the PMSs of all name components. SNMS_tr_ is a non-linear transformation of SNMS, scaled to provide a more intuitive measure of the confidence that a submitted string matches a name. SNMS_tr_ , which ranges from 0-1, where 1 is a perfect match, is tolerant of variation in SNMS when the submitted name has a very good match or no match, but sensitive to small differences in the middle of the range of SNMS. This reflects the intuitive perception that it takes more evidence to change an opinion when one has very high confidence that it is correct than when one is uncertain. SNMS_tr_ is an arctangent transformation of SNMS, normalized by the number of name components:

(2) SNMStr=atans*SNMS/k^2*t+1/2*atans^2*t+1+0.5

where k is the number of name components, *s* > 0 and *t* ≥ 0 are two parameters that change the shape of the transformation, from a linear relationship (*s*≈0, t=0), to different forms of logistic (s > 1, t = 0) and double-logistic functions (s > 1, t > 1). The parameter *s* > 1 can be used to control the steepness of the curve, whereas *t* > 1 controls the size of the center. The TNRS uses values of *s* = 2 and t = 1. This configuration divides the curve in 5 regions: 2 regions of certainty at the two extremes, a central region of uncertainty and 2 regions of discrimination that fall in between (Figure 1). In the regions of certainty and in the central region, differences in SNMS produce only small changes in SNMS_tr_ whereas in the regions of discrimination, small differences in SNMS are amplified by the transformation.

The fourth and final step in the calculation of OMS takes into account the authority and unmatched name components, if any. A fixed penalty of 0.1 is subtracted if any unmatched text was found that did not matched to a name, an author, or a standard annotation such as "cf.". If an author was submitted, the OMS is calculated as a weighted average of the SNMS_tr_ and the AMS. The TNRS is implemented with 0.8 and 0.2 as the weights for the SNMS_tr_ and AMS, respectively. Thus, for a name plus author,

(3) $$\mathrm{OMS}=\left(0.8*{\mathrm{SNMS}}_{\mathrm{tr}}\right)+\left(0.2*\mathrm{AMS}\right)-p$$

where p is a penalty which equals 0.1 if unmatched text was found, otherwise 0. If no author was submitted,

(4) $$\mathrm{OMS}={\mathrm{SNMS}}_{\mathrm{tr}}-p$$

This is the final OMS that is presented to the user.

***Candidate match selection.*** To qualify as a candidate match, a name must pass the maximum ED test and also pass either the phonetic test or the match threshold test. The phonetic test is performed during fuzzy matching (see ***Fuzzy matching***, above). The remaining two tests are performed during post processing, as described below.

To pass the maximum ED test, the following must be true: ED ≤ 2 * (number of name parts). Rank indicators of infraspecific taxon names ("var.", "subsp.", etc.) are not counted as name parts. Thus, for a variety such as *Poa annua* var. *spuria*, the number of name parts is three, and the maximum ED is 6. For a species name, which consists of two parts, the maximum ED is 4.

The match threshold test is based on the EDs of each name component, weighted by the lengths of the strings compared. The following conditions must be satisfied for each name component: (ED / MSL ≤ MaxEDR) AND ((2 ≤ ED < 4 AND the first character matches) OR (ED = 4 AND the first 3 characters match)), where MSL is the minimum length of the two strings being compared and MaxEDR is the maximum edit distance ratio, a constant which takes on one of two values depending on the value of MSL. For MSL < 6, MaxEDR= 0.5; for MSL ≥ 6, MaxEDR= 0.3334. The values of MaxEDR were determined empirically by examining performance for samples of names. Although in general MaxEDR=1/3 provides intuitively "reasonable" matching of most names (BB, pers. observation), it is increased for strings of five characters or less to compensate for a bias against matching short strings. For example, "Marsilleya", which differs from its target genus "Marsilea" by an ED of 2 (MSL=8), passes the match threshold test (ED/MSL ≤ 0.3334). "Ulleya", which also differs from its target "Ulea" by an ED of 2 (MSL=4), passes the match threshold test at the less stringent MaxEDR of 0.5 (ED/MSL ≤ 0.5), but would fail at MaxEDR=0.3334.

***Ranking and best match selection.*** Once candidate matches have been determined by applying the phonetic, match threshold and maximum ED tests, multiple candidate matches to a single submitted name are ranked to select the best match. During name processing, the TNRS performs and stores two alternative sets of rankings, one unconstrained and the other constrained by higher taxonomy.

Using the unconstrained algorithm, the TNRS ranks all candidate matches by descending SNMS, OMS and taxonomic status. Taxonomic status is ranked as follows: “accepted” > “synonym” > “no opinion” ("illegitimate" and "invalid" are treated as "synonym" for ranking purposes). The highest ranking candidate match is then presented to the user as the best match. If two or more candidate matches have identical values of SNMS, OMS and taxonomic status, the name with the lowest alphabetical sort order is presented as the best match but flagged as “Ambiguous Match” (see **Warnings**, below).

The taxonomically-constrained rank calculation is similar to the unconstrained algorithm, except that the calculation is performed separately for each name component, starting with genus (or family, if a family was submitted with the name), then species, then infraspecific taxa, if any. The result of the taxonomically-constrained algorithm is that the best (highest ranked) match for a species name with a misspelled genus but perfectly spelled specific epithet will be the best-matching genus, whereas the best match using the unconstrained algorithm will be best-matching species. For example, the best overall match for *Fucus insipida* is the species *Ficus insipida* (OMS = 0.96) in the Moraceae (fig family), whereas the best genus match is *Fucus* (OMS = 0.50) in the Fucaceae (brown algae). Under the default unconstrained ranking algorithm, *Ficus insipida* will be displayed as the best match and flagged with the warning "Better higher taxonomic match available". Under the taxonomically-constrained ranking algorithm, *Fucus* will be displayed as the best match and given two warnings: "Partial match" and "Better spelling match in different higher taxon" (see **Warnings**, below).

Both sets of rankings are calculated and stored during name processing, and the user may switch between taxonomically-constrained or unconstrained matches after name processing is complete by checking or unchecking "Constrain by higher taxonomy" under "Best match settings" (see **User options**). In addition, a second user setting provides the option of constraining best matches by taxonomic source. This ranking is performed "on the fly" by checking "Constrain by source" under "Best match settings", and causes all candidate matches from the top-ranked source to rank above those of lower-ranked sources. Thus, a candidate match with a low OMS will appear as the best match even if candidate match with a higher OMS is found for a lower-ranked taxonomic source. Adjusting this setting only has an effect if >1 taxonomic source has been used. This setting is recommended if using multiple sources simultaneously, as it minimizes the effect of spelling and synonymy conflicts between sources.

***Warnings.*** The TNRS issues four types of warnings about names matched. "Partial match" indicates that the name matched is of a higher taxonomic rank than the name submitted by the user. For example, if the user submitted a species name but the TNRS was able to match only the genus, the TNRS would return the genus along with the warning "Partial match". "Ambiguous match" indicates a “tie”, meaning that one or more other candidate matches have identical match scores and taxonomic status. Two additional warnings indicate that the name submitted matches to taxa which are not closely related. "Better spelling match in different higher taxon" indicates that another candidate match with a better overall match score is available in a different higher taxon. "Better higher taxonomic match available" indicates that a another candidate match with a lower overall match score provides a better match to the higher taxon of the name submitted (see ***Ranking and best match selection***, above).

#### Web services and application programming interface

The TNRS web-services layer acts as an asynchronous job execution and data management server. It controls traffic between the user interface and the TNRS name resolution. These services manage input and output files and schedule jobs submitted for parsing and fuzzy matching.

The TNRS web services can be accessed programmatically via a RESTful API, using a GET call with two parameters: *retrieve* (followed by options requesting the return of all matches or the single best match only) and *names* (followed by a comma-separated list of URL-encoded taxon names). Results are returned from the web service as JSON. Details of the TNRS API are provided at http://tnrs.iplantcollaborative.org/about.html#api . As a demonstation of how to use the TNRS in third-party software, Additional file 1 provides an example R script that calls the TNRS API in the context of adding taxon names to a phylogeny.

#### Web interface

The TNRS web interface uses a Rich Internet Application (RIA) [65] front end and is built using the Google Web Toolkit for a high degree of user interactivity within a web browser. The interface is supported by a layer of web services that provide a bridge between the user interface (UI) and the underlying algorithms that perform the matching (see ***Web services and application programming interface***).

**Figure 2:**
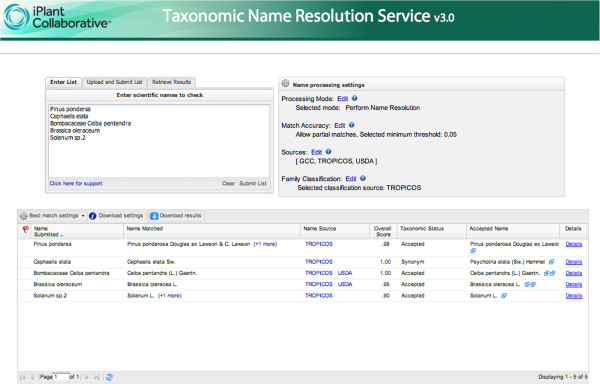
**Screenshot of the main TNRS user interface.** Up to 5000 names, one per line, may be entered manually or pasted into the “Enter list” text box. Larger lists are uploaded using the "Upload and Submit List" tab. Name processing settings are adjusted prior to submitting the names using the controls in the upper left box. Best match settings, on the upper left of the results display, are set after results are returned, and affect how multiple results for the same name are ranked and therefore how the single best match is selected. The "(+n more)" link allows the user to view and select any alternative matches found. The "Details" hyperlink displays the results and match scores for each name component (genus, species, author, etc.). The remaining hyperlinks link to entries in the original source databases. "Download settings" displays a report of all settings used to resolve the current batch of names. The "Download results" button displays options for downloading results as a plain text file.

The TNRS web interface allows the user to submit names in a text box or by uploading a file (Figure 2). Names submitted via the file load utility may also be preceded by an integer ID separated by a tab. Including a numeric ID provides an alternative way of joining results back to the original database.

Name resolution results are displayed below the data entry panel (Figure 2). Only the single best match is displayed by the web interface; however, users may view alternative matches by clicking on the “(+*n* more)” hyperlink. Additional hyperlinks allow the user to view matched names and accepted names in their original source databases. Taxonomic status of each name is indicated as “Accepted”, “Synonym” or “No opinion” (some sources further distinguish "Illegitimate" or "Invalid" non-accepted names). For matched names that are not accepted according to the sources consulted a link to the accepted name is provided. Results can be copied directly from the results display or downloaded as a comma-delimited text file.

***User options.*** Users can configure two types of options: Name processing settings and best match settings. Name processing settings must be adjusted prior to submitting names for processing, and are displayed to the right of the data entry panel (Figure 2; see Table 2 for details). Best match settings affect how the best match is selected by adjusting the algorithm used to rank multiple candidate matches (see ***Ranking and best match selection***). These settings are adjusted "on the fly" after names have be processed by the TNRS, and are displayed in a drop-down menu on the upper left of the results display (Figure 2). Changes in best match settings are reflected immediately in the display and in the downloaded results file.

**Table 2:** Name processing settings

**Setting**	**Description**	**Options**
Processing mode	Determines whether the name is parsed and resolved (corrected) or parsed only	Full name resolution (default)
		Parse names only
Match accuracy	Adjusts the minimum OMS required to return a name as a candidate match	Slider from lowest (default) to highest (perfect match, OMS = 1.0)
Allow partial matches	If enabled, the TNRS will match a higher taxonomic component of a name if it cannot match the name at the rank submitted	Enabled (default)
		Not enabled
Sources	Taxonomic sources used to resolve names. Higher-ranked sources applied first if Best match setting "Constrain by source" enabled (see text)	Select
		Deselect
		Rank by dragging/dropping
Family classification	Source of family classification for matched and accepted names	Tropicos / APG III (default)
		NCBI (similar to APG III, with recent changes)

These user options must be set prior to submitting names for processing. Best match settings (not listed; see User options) are adjusted after processing is complete.

## Results and discussion

### Performance evaluation

#### Comparison with existing name resolution applications

Although some the functionality of the TNRS can be found within existing name resolution applications, none combine all the capabilities of fuzzy matching, synonym correction, partial matching, return of alternative matches, and homonym resolution within both an API and user-friendly web interface (Table 3). Web services such as the Tropicos web service [66] and Catalogue of Life [33] are capable of bulk resolution of plant names but do not use fuzzy matching to correct misspelled names or standardize variant spellings. The Tropicos batch name matching utility [67] performs exact matching but does not correct misspelled names. The Taxamatch implementation used by the Interim Register of Marine and Nonmarine Genera (IRMNG) [64] performs fuzzy matching but does not currently handle infraspecific taxa or perform batch correction of misspelled names. The GRIN Taxonomic Nomenclature Checker (GRIN-TNC) [68], an important early name resolution application, performs fuzzy matching based on Levenshtein EDs and is capable of bulk name resolution; however, resolution must be done in stages by first correcting genera, then resubmitting species. The GRIN-TNC does not provide confidence scores or alternative matches and is available only via a web user interface.

**Table 3:** Comparison of features of name resolution applications

**Application**	**Batch processing**	**Fuzzy matching**	**Corrects synonyms**	**Provides confidence score**	**Returns alternative matches**	**API**	**User interface**	**Handles infraspecific taxa**
TNRS	x	x	x	x	x	x	x	x
Tropicos web service	x		x			x		x
Catalogue of Life	x		x				x	x
Tropicos name matching utility	x						x	x
Taxamatch (IRMNG)		x	x	x	x		x	
GNResolver	x	x	x	x	x	x	x	x
GRIN Taxonomic Nomenclature Checker	x	x	x				x	x
Plantminer	x	x	x			x	x	x

To our knowledge, Plantminer [69] and The Global Names Resolver (GNResolver) [70] are the only applications in addition to the TNRS that combine batch resolution of plant scientific names, spelling correction via fuzzy matching, and access via both a user interface and web services. Like the TNRS, both these applications convert synonyms to accepted names. Like the TNRS (but unlike Plantminer) the GNResolver can provide alternative matches and also returns a score indicating overall level of confidence in the match. To compare the name matching abilities of Plantminer and the GNResolver relative to the TNRS, we submitted to each application a list of 1000 uncorrected plant names from a database of ecological inventories (The SALVIAS Project [9]; see Additional file 2). The list contained a variety of errors such as misspelled taxon names, annotations, frame shifts, unconverted extended ASCII codes, morphospecies, etc. For the TNRS, we used Tropicos as the only taxonomic source; all other options were left at the default settings. As Plantminer checks names against both The Plant List and Tropicos, we expected that all names resolved by the TNRS should also be discoverable by Plantminer. Tropicos taxonomy is not available for use by the GNResolver; instead, we selected the International Plant Names Index (IPNI) [30] as the taxonomic source due to the high overlap between the two databases [71]. We scored a name as successfully resolved if the application returned the expected name, as determined by inspection against the Tropicos database. For the GNResolver, we excluded from error counts any names which failed to resolve because the intended name was not in IPNI. Due to different conventions for spelling and abbreviation of author names between the source databases, we did not require matching of authorities.

The TNRS processed the 1000 names in 43 sec, or 0.04 sec/name, successfully correcting 980 names. Of the 20 failed matches, 17 were incomplete (matching to genus only), one was a non-match, and two were incorrect matches (matches to the wrong name). Plantminer processed the names in 10 min 13 sec, or 0.6 sec/name, successfully correcting 881 names. Of the 119 failed matches, 20 were incomplete, 76 were non-matches, and 23 were incorrect matches. The GNResolver processed the names in 5 min 12 sec or 0.3 sec/name successfully correcting 745 names. Of the 255 failed matches, 33 were incomplete, 226 were non-matches, and 4 were incorrect matches.

Most errors made by the TNRS (13 names, 65% of total errors) were incomplete matches due to badly-misspelled names outside the match threshold (Table 4; see Additional file 3 for a complete list of all incorrectly matched names). The remaining failures were caused by numbers in the authority, capitalized specific epithets, and mistaking morphospecies names or non-standard annotations for scientific name components (for example, the second part of the variant annotation "sp. nova" ("sp. nov." or new species) was converted to the specific epithet "nana"). The largest category of name resolution errors by Plantminer (58 names, 48%) were due to failure to recognize the standard annotations "cf.", "aff." and the non-standard but commonly-used "indet.". In most cases the presence of these terms resulted in non-matches rather than partial matches. Also common was failing to make a partial match (35 names, 19 to family and 15 to genus) for names accompanied by annotations or morphospecies strings (e.g., "Fabaceae Indet. sp. 21" was correctly matched to "Fabaceae" by the TNRS, but was not matched by Plantminer). By far the largest source of error for the GNResolver (217 names, 85% of total erroneous names) were non-matches caused by names in all capital letters. Even when perfectly spelled, such names resulted in non-matches. Other major causes of error were misspelled names outside the match threshold, failure to recognize some annotations (in particular "aff." and embedded question marks), and parsing errors triggered by special characters such as pipe ("|") in the name submitted.

**Table 4:** Types of errors made during resolution of 1000 names by Plantminer, GNResolver and the TNRS

**Most likely cause of error**	**Plantminer**	**GNResolver**	**TNRS**
Annotation not recognized	58	21	3
Name all caps		217	
Capitalized specific epithet		1	1
Failed to match family or genus	34		
Infraspecific rank indicator not recognized	3		
Morphospecies treated as taxon	15		1
Name submitted matches to >1 name	8	4	
Failed fuzzy match, outside threshold		9	13
Parsing error caused by number in authority			2
Parsing error caused by special character in name		2	
Unknown	1	1	
Total	119	255	20

Features of the TNRS that enabled it to achieve a higher rate of success than both Plantminer and the GNResolver included recognition of a larger diversity of botanical annotations and alternative formulations of infraspecific rank indicators ("ssp" instead of "subsp."), the ability to perform partial matches to genus or family when the full name cannot be matched, and reduced sensitivity to case. In addition, the above results suggest that most TNRS match failures are easily remedied by allowing a less strict match threshold than the current default (although at the risk of an increased rate of false positives). Finally, this test compared only the abilities of the three applications to match names. Features such as warning flags, constraining by higher taxonomy, and tools for comparing and selecting alternative matches are unique to the TNRS and cannot be compared to other applications.

#### Improving linkages between taxonomic databases

As a test of the ability of the TNRS to increase linkages among biodiversity datasets, we compared overlap between two major taxonomic databases, pre- and post-standardization with the TNRS. The databases compared were the Integrated Taxonomic Information System (ITIS [45]) and the National Center for Biotechnology Information taxonomic database (NCBI, the taxonomic component of GenBank [72]). From each database, we extracted all plant names at the rank of species or below (NCBI, viridiplantae subtree; ITIS, kingdom="Plantae"). From NCBI, we included only formal scientific names, excluding informal names referring to samples or accessions (so-called “dark taxa” *sensu* R. Page [47]).

The lists of unique names from both databases combined were then standardized using the TNRS. As both USDA Plants and NCBI can be used as taxonomic sources by the TNRS (USDA species are in theory a subset of those in ITIS), we used only Tropicos and the GCC (Global Compositae Checklist) as taxonomic sources. All other options were left at their default settings.

Prior to standardization, 4,412 names out of a combined total of 141,814 (roughly 3%) were shared between the two databases. After standardization and matching of names by the TNRS, plus conversion of synonyms, total names dropped to 114,497 and the overlap between the two databases increased to 20,670, or 18% (Table 5). Interestingly, much of the gain in overlap (or conversely, loss of superfluous or incorrect names) occurred during matching (a 350% increase), rather than conversion of synonyms (an additional 16% increase; see Table 5). Overall, at least 27,317 names in the combined databases, or 19.2%, were erroneous or redundant entries due to spelling errors, variant spellings or synonymy.

**Table 5:** Total names within two plant taxonomic databases before and after name resolution using the TNRS

**Name source**	**Original names**	**After matching by TNRS**	**After matching & synonym conversion by TNRS**
NCBI	99743	97734	90142
ITIS	46483	45960	45025
NCBI+ITIS (shared names)	4412	19935	20670
NCBI+ITIS (total unique names)	141814	123759	114497

Totals after matching include the original name if no match was found by the TNRS. Totals after matching and synonym conversion use accepted names in place of synonymous matched names.

The most important outcome of name resolution was the nearly five-fold increase in taxonomic overlap between the two taxonomic databases. This result highlights the potential of taxonomic resolution as a general tool for integrating and building linkages between biodiversity databases.

### Future directions

One of the primary strengths of the TNRS is to provide a repeatable and efficient workflow for accessing existing, best available taxonomic sources. The ease with which new sources are added to the TNRS database suggest that future efforts should be directed to encouraging providers of high-quality taxonomy to make their information available via the TNRS.

Although the TNRS provides a way to resolve many common forms of taxonomic semantic heterogeneity—in particular ambiguities due to misspellings and lexical variants, nomenclatural synonyms, and many forms of homonyms—major challenges remain. In particular, divergent taxonomic concepts can translate to differences in traits and geographic distributions; yet such differences are not reflected by differences in taxon names and authorities [39]. For example, depending on the concept used, *Abies lasiocarpa* (Hook.) Nutt. (subalpine fir) is either (a) widely distributed throughout the Pacific Northwest and the interior Rocky Mountains of North America or (b) restricted to the coastal ranges of British Columbia and Alaska. A more complex example is provided by the grass *Andropogon virginicus* where the name has at least 5 meanings that overlap with 17 different taxon concepts that are variously given 27 scientific names [73]. Unfortunately, at the present time, disambiguating such taxonomic ambiguity due to differing taxon concepts requires information on usage not communicated by the name alone, and rarely provided by most current taxonomic sources (but see [73]). As such information becomes available, future efforts should be directed toward the resolution not simply of names but of biologically more meaningful taxon concepts.

Although the TNRS was developed to resolve plant names, relatively minor changes are needed to extend coverage to other organisms and nomenclatural codes. Such improvements are beyond the scope of the current project, but we encourage others in the community to adapt the TNRS to their needs by accessing the source code at our publicly available repository.

## Conclusions

The increasing availability of large, digitized biological datasets, while clearly a boon for biodiversity research, is also leading to an accumulation of incorrect, ambiguous or outdated taxon names, with negative consequences for comparative biological science, policy making, and data discovery. In an effort to provide a way forward we have developed the Taxonomic Name Resolution Service or TNRS, an application for correcting and standardizing taxonomic names with reference to existing sources of high-quality taxonomy.

The TNRS combines, within a single application, automated name parsing and correction with tools for inspection and resolution of ambiguous results. The TNRS provides a labor-saving and repeatable workflow for standardizing taxonomic names across an array of legacy and contemporary biodiversity data. A web interface makes the TNRS accessible to non-specialist users, while web services support programmatic access by expert users in need of automated name resolution. Tests demonstrate the potential of the TNRS for reducing error and increasing integration among major organismal databases.

## Availability and requirements

**Project name:** Taxonomic Name Resolution Service

**Project home page:**http://tnrs.iplantcollaborative.org/

**Operating systems:** Linux based

**Programming languages:** PHP, MySQL, Ruby, Java

**Other requirements:** Java JDK 1.7.0 or higher, Git 1.7.4 or higher, MySQL 5.0.95 or higher, PHP 5.3.3 or higher (including mysql and mbstring extensions), Maven 2.2.1 or higher, Apache Tomcat 7.0.33 or higher, Apache HTTP Server 2.2.3 or higher, Apache JK Modules 1.2.31 or higher, YAML 0.1.4, Ruby 1.9.3 or higher, Rubygems 1.8.23 or higher. The setup has been tested on CentOS 5.8. Details are available at http://github.com/iPlantCollaborativeOpenSource/TNRS/blob/master/INSTALL.

**Licences:** The TNRS was built on two existing open-source projects, each of which retain their original licensing. The SilverBiology PHP port of Taxamatch [56] uses the Apache 2.0 license, and GNI's name parser uses a BSD style license. All other code is licensed using a standard BSD license [74].

**Any restrictions to use by non-academics:** None

**Access to source code:** The TNRS user interface is freely accessible via the TNRS website at http://tnrs.iplantcollaborative.org/. Instructions for accessing the TNRS matchNames web service can be found at http://tnrs.iplantcollaborative.org/api.html. Developers wishing to modify the TNRS for their own needs can download source code from the iPlant GitHub repository at https://github.com/iPlantCollaborativeOpenSource/TNRS/. A virtual machine image of the TNRS pre-loaded with an example database can be launched from within iPlant's Atmosphere cloud computing environment (https://atmo.iplantcollaborative.org; requires iPlant credentials).

## Abbreviations

AMS: Author match score; API: Application programming interface; ED: Edit distance; MaxEDR: Maximum edit distance ratio; MSL: Minimum length of the two strings being compared; OMS: Overall match score; PMS: Partial match score; SNMS: Scientific name match score; SNMS_tr_: Transformed scientific name match score.

## Competing interests

The authors declare that they have no competing interests.

## Authors’ contributions

Initial concept for the TNRS was developed by BB and BJE, with later suggestions from WHP, RKP, ZL, JARG, CF, SJM, NM, MN and NH. BB, CF, WP and RKP participated in the preliminary TNRS planning meeting at the Missouri Botanical Garden. The GNI Parser was designed by DM. Taxamatch was conceived and developed as a standalone product by TR, who assisted with advice regarding its implementation within the TNRS. The core database and database loading scripts were developed by BB, with assistance from ZL and TR. Pre- and post-processing code and Taxamatch extensions were written by ZL. User interface and web services layer were coded by JARG. Scoring and ranking algorithms were developed by TR, DM, ZL, BB and NM. Project direction was provided by NH, MN and SL. The initial draft of the paper was written by BB. All authors read, participated in revision, and agreed with the final manuscript.

## Supplementary Material

Additional file 1 Example R script which uses the TNRS API to correct names on a phylogeny. Click here for file

Additional file 2 Taxonomic names used to compare performance of TNRS, Plantminer and GNResolver. Click here for file

Additional file 3 List of submitted names, expected targets, and descriptions of errors for names which failed matching by one or more name resolution applications. Click here for file
